# Downregulation of Placental Amino Acid Transporter Expression and mTORC1 Signaling Activity Contributes to Fetal Growth Retardation in Diabetic Rats

**DOI:** 10.3390/ijms21051849

**Published:** 2020-03-07

**Authors:** Jie Xu, Jiao Wang, Yang Cao, Xiaotong Jia, Yujia Huang, Minghui Cai, Chunmei Lu, Hui Zhu

**Affiliations:** 1 Department of Physiology, Harbin Medical University, Harbin 150081, China; xujie@ems.hrbmu.edu.cn (J.X.); wangj_84321@163.com (J.W.); caoyanghmu@163.com (Y.C.); jiaqiaoran@icloud.com (X.J.); woainimen924@sina.com (Y.H.); caiminghui@hrbmu.edu.cn (M.C.); 2 Laboratory of Medical Genetics, Harbin Medical University, and The Key Laboratory of Preservation of Human Genetic Resources and Disease Control in China, Chinese Ministry of Education, Harbin 150081, China

**Keywords:** placenta, amino acid transporter, mammalian target of rapamycin, gestational diabetes

## Abstract

Alterations in placental transport may contribute to abnormal fetal intrauterine growth in pregnancies complicated by diabetes, but it is not clear whether the placental amino acid transport system is altered in diabetic pregnancies. We therefore studied the changes in the expressions of placental amino acid transporters in a rat model of diabetes induced by streptozotocin, and tested the effects of hyperglycemia on trophoblast amino acid transporter in vitro. Our results showed that the expressions for key isoforms of system L amino acid transporters were significantly reduced in the placentas of streptozotocin-induced diabetic pregnant rats, which was associated with the decreased birthweight in the rats. A decreased placental efficiency and decreased placental mammalian target of rapamycin (mTOR) complex 1 (mTORC1) activity were also found in the rat model. In addition, hyperglycemia in vitro could inhibit amino acid transporter expression and mTORC1 activity in human trophoblast. Inhibition of mTORC1 activity led to reduced amino acid transporter expression in placental trophoblast. We concluded that reduced placental mTORC1 activity during pregnancy resulted in decreased placental amino acid transporter expression and, subsequently, contributed to fetal intrauterine growth restriction in pregnancies complicated with diabetes.

## 1. Introduction

Diabetes in pregnancy is considered a high-risk condition for maternal and neonatal morbidity and remains a significant medical challenge. Women with diabetes have a higher risk of preeclampsia, caesarean section, and fetal outcomes including congenital anomalies, stillbirth, and macrosomia. Moreover, women with diabetes at the time of conception have more adverse outcomes (i.e., birth defects, perinatal mortality, and morbidity) than women who develop gestational diabetes during pregnancy [1]. The adverse outcomes associated with diabetes in pregnancy are substantially associated with hyperglycemia. However, the molecular mechanism underlying how maternal hyperglycemia leads to abnormal fetal growth remains unclear.

Placental transfer of amino acids via amino acid transporters is essential for optimal fetal growth and development. Decrease in amino acid transport by the placenta is implicated as a potential cause of fetal growth restriction. There are a variety of such transporters, selective for various classes of amino acids. Na^+^-dependent system A amino acid transporter is specific for neutral amino acids with short side chains such as glycine and alanine. System L amino acid transporter is Na^+^-independent exchanger specific for large neutral amino acids. Studies have shown that placental system A activity is lower in intrauterine growth restriction (IUGR) compared with normal pregnancies [2,3]. However, alterations in the placental amino acid transporters in pregnancies complicated by diabetes are largely unknown.

The mammalian target of rapamycin (mTOR) signaling pathway has newly been suggested to be a nutrient sensor in the placenta. mTOR is a ubiquitously expressed serine/threonine protein kinase that exists in two complexes, mTOR complexes 1 (mTORC1) and 2 (mTORC2). Placental nutrient transporter expression is mainly regulated by mTORC1. We previously reported that mTORC1 might regulate placental glucose transport by altering the glucose transporter isoform-3 expression in placental trophoblast [4]. In addition, evidence has shown that mTORC1 could regulate placental amino acid transport by modulating the cell surface abundance of system A and system L transporter isoforms without affecting global protein expression in primary human trophoblast cells [5]. Alterations of placental mTORC1 activity has been found as being associated with adverse pregnancy outcomes. For example, studies have shown that placental mTORC1 activity was decreased in human IUGR [6] or animal model of IUGR [7], suggesting placental mTORC1 plays an important role in the development of abnormal fetal growth. Although dysregulation of placental mTOR has been well documented in IUGR pregnancies, it has rarely been studied in pregnancy complicated with diabetes.

It is speculated that mTORC1 may be activated in diabetes in association with increased placental amino acid transport [8]. Thus, we aimed to investigate the alteration of mTORC1 pathway and system L amino acid transporters in diabetic placentas and to study the impacts of high glucose on mTORC1 activity and system L amino acid transporter expression in placental trophoblast cells.

## 2. Results

### 2.1. STZ Induced Severe Diabetes in Rats

**Figure 1:**
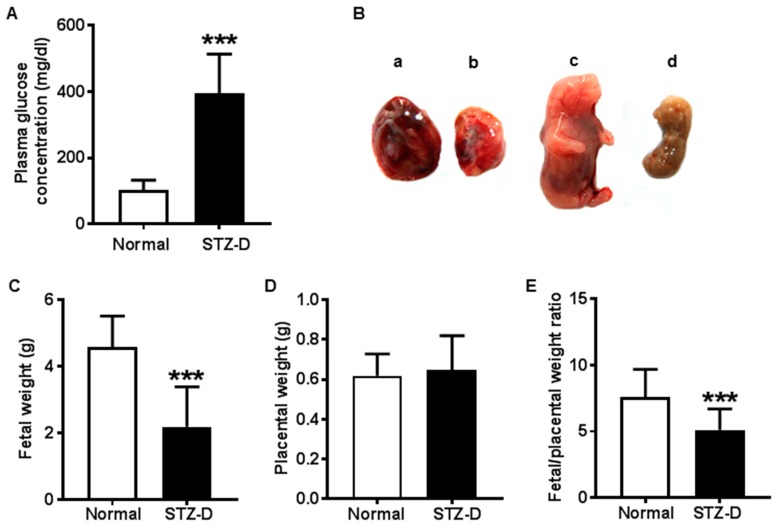
Pregestational diabetes led to fetal growth restriction and decreased placental efficiency in streptozotocin-induced diabetes (STZ-D) rats. (**A**) Maternal plasma glucose levels of normal and STZ-D rats (*n* = 13). (**B**) Representative pictures of abnormal growth of the fetus and placentas in STZ-D rats: a, normal placenta; b, STZ-D placenta; c, normal fetus; d, STZ-D fetus. (**C**) Birthweight from normal and STZ-D rats, showing that STZ-D pregnant rats had decreased birthweight compared with normal rats (*n* = 30). (**D**) Placental weight derived from normal and STZ-D rats (*n* = 30). (**E**) Fetal weight/placental weight ratio (*n* = 30) showing that STZ-D rats had decreased fetal to placental weight ratio. Data are expressed as mean ± SD. *** *p* < 0.001, normal versus STZ-D.

Maternal glucose concentration was markedly increased in the rats injected with streptozotocin (STZ). All the rats developed severe diabetes according to their blood glucose levels (greater than 300 mg/dl) in the STZ-induced diabetes (STZ-D) group (Figure 1A), and the maternal rats showed classical features of type 1 diabetes, such as polyphagia, polyuria, and polydipsia (data not shown). In addition, maternal diabetes resulted in adverse placental and fetal outcomes, as shown in Figure 1B.

### 2.2. Pregestational Diabetes Resulted in Fetal Growth Restriction and Decreased Placental Efficiency in Rats

Fetal weight was significantly decreased in STZ-D rats compared with normal rats, suggesting newborns from severe diabetic mothers presented intrauterine growth restriction (Figure 1C). Nonetheless, there was no significant difference in placental weight (Figure 1D). However, the fetal weight/placental weight ratio was significantly decreased in STZ-D rats compared with normal rats (Figure 1E). The data are summarized in Table 1.

Because the fetal weight/placental weight ratio was considered as an indicator of placental efficiency, our results suggested that severe pregestational diabetes led to decreased placental efficiency, which might contribute to fetal growth delay in diabetic pregnancies.

### 2.3. Pregestational Diabetes Resulted in Decreased Placental Amino Acid Transporter Expression in Rats

**Figure 2:**
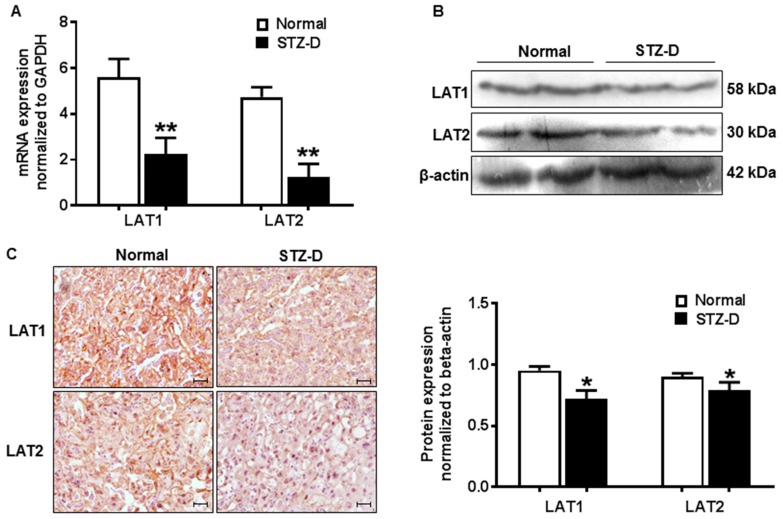
Pregestational diabetes resulted in decreased placental amino acid transporter expression in STZ-D rats. (**A**) Relative mRNA expression of system L amino acid transporter LAT1 and LAT2 detected by quantitative-PCR in placentas derived from normal and STZ-D pregnant rats (*n* = 3). (**B**) Expression of LAT1 and LAT2 detected by Western blots in placentas from normal and STZ-D rats. The bar graph shows the relative density of protein expression for LAT1 and LAT2 after normalization with β-actin expression in each sample. Data are mean ± SD from six normal and six STZ-D placentas. * *p* < 0.05, ** *p* < 0.01, normal versus STZ-D. (**C**) Representative immunostaining images of LAT1 and LAT2 expressions in tissue sections from normal and STZ-D placentas. Scale bar, 250 μm.

Expression of placental system L amino acid transporter isoform LAT1 and LAT2 was assayed by quantitative-PCR and Western blot in normal and diabetic pregnant rats. Our results showed that relative mRNA expression of placental LAT1 and LAT2 was significantly reduced in STZ-D rats compared with normal pregnancies (Figure 2A). Protein level of placental LAT1 (0.95 ± 0.03 vs. 0.72 ± 0.07, normal vs. STZ-D, *p* < 0.05) and LAT2 (0.90 ± 0.03 vs. 0.79 ± 0.06, normal vs. STZ-D, *p* < 0.05) were also decreased in STZ-D pregnant rats (Figure 2B). LAT1 and LAT2 expression was also examined by immunohistochemistry staining in placental tissue sections. Consistent with Western blot data, pregestational diabetes caused reduction of LAT1 and LAT2 expression in the placentas (Figure 2C). These findings indicated that down-regulation of placental amino acid transporters was closely associated with fetal growth restriction and decreased placental efficiency in the rat model of severe gestational diabetes.

### 2.4. Pregestational Diabetes Reduced Placental mTORC1 Activity in Rats

**Figure 3:**
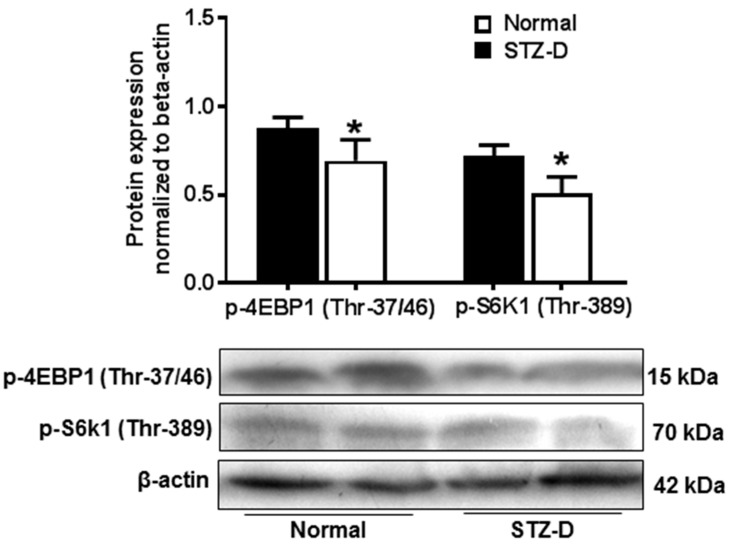
Placental mammalian target of rapamycin (mTOR) complex 1 (mTORC1) activity was decreased in STZ-D rats. Protein expression of p-4E-bingding protein 1 (4EBP1) and p-S6 kinase1 (S6K1) was detected by Western blot in placentas from normal and STZ-D pregnant rats. The bar graphs show relative expression after being normalized with β-actin expression in each sample. Gestational diabetes induced a decrease in p-4EBP1 and p-S6K1 expression. The lower panel shows representative blots for p-4EBP1 and p-S6K1. * *p* < 0.05, normal versus STZ-D. Data are from six independent experiments.

To investigate the alterations of placental mTORC1 activity in diabetic pregnancies, expressions of phosphorylated S6 kinase1 (p-S6K1) and eukaryotic translation initiation factor 4E-bingding protein 1 (p-4EBP1), two down-stream regulators of mTORC1, were examined in normal and STZ-D placentas. As shown in Figure 3, placental p-4EBP1(Thr-37/46) (0.87 ± 0.06 vs. 0.69 ± 0.12, normal vs. STZ-D, *p* < 0.05) and p-S6k1(Thr-389) (0.72 ± 0.06 vs. 0.51 ± 0.09, normal vs. STZ-D, *p* < 0.05) expressions were significantly reduced in STZ-D rats compared with normal rats, which suggested that severe pregestational diabetes could decrease placental mTORC1 signaling activity.

### 2.5. Hyperglycemia in Vitro Down-Regulated Amino Acid Transporter Expression and mTORC1 Activity in JEG-3 Trophoblast Cells

**Figure 4:**
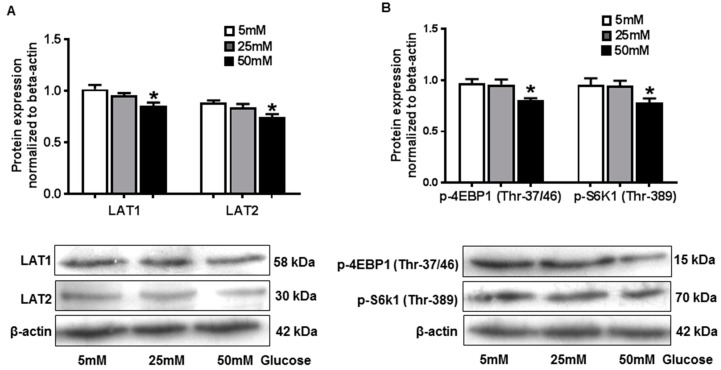
High glucose in vitro down-regulated amino acid transporter expression and mTORC1 activity in JEG-3 trophoblast cells. (**A**) Protein expression of LAT1 and LAT2 detected by Western blot in JEG-3 trophoblast cells cultured with different concentration of glucose. The bar graphs show relative expression after being normalized with β-actin expression in each sample. We found that 50mM glucose induced a significant decrease in LAT1 and LAT2 expression. The lower panel shows representative blots for LAT1 and LAT2. * *p* < 0.05, 5mM versus 50mM. Data are from five independent experiments. (**B**) Protein expression of p-4EBP1 and p-S6K1 detected by Western blot in JEG-3 trophoblast cells cultured with different concentration of glucose. The bar graphs show relative expression after being normalized with β-actin expression in each sample. We found that 50 mM glucose induced a significant decrease in p-4EBP1 and p-S6K1 expression. The lower panel shows representative blots for p-4EBP1 and p-S6K1. * *p* < 0.05, 5 mM versus 50 mM. Data are from five independent experiments.

To study the regulation of hyperglycemia on placental amino acid transporter and mTORC1 activity in vitro, human trophoblast cell line JEG-3 was cultured with different concentrations of glucose, and expression of LAT1, LAT2, p-4EBP1(Thr-37/46), and p-S6k1(Thr-389) was examined by Western blot. Our results showed that LAT1 (1.02 ± 0.09 vs. 0.86 ± 0.06, 5 mM vs. 50 mM, *p* < 0.05) and LAT2 (0.89 ± 0.05 vs. 0.75 ± 0.06, 5 mM vs. 50 mM, *p* < 0.05) expression was significantly decreased in JEG-3 cells treated with 50 mM glucose compared to the cells cultured with 5 mM glucose (Figure 4A), which indicated the fact that high glucose would attenuate amino acid transporter expression in trophoblast cells. p-4EBP1(Thr-37/46) (0.97 ± 0.09 vs. 0.80 ± 0.05, 5 mM vs. 50 mM, *p* < 0.05) and p-S6k1 (Thr-389) (0.95 ± 0.14 vs. 0.78 ± 0.08, 5 mM vs. 50 mM, *p* < 0.05) expression was also reduced in cells treated with 50mM glucose compared with the cells cultured with 5mM glucose (Figure 4B), suggesting high glucose in vitro could inhibit mTORC1 activity in placental trophoblast cells. Together with the findings in the STZ-D rats, our data suggest that maternal diabetes could lead to decreased placental amino acid transporter expression and mTORC1 activity, which may contribute to the fetal intrauterine growth restriction in diabetic pregnancies.

### 2.6. Inhibition of mTORC1 Activity Resulted in Decreased Amino Acid Transporter Expression in JEG-3 Trophoblast Cells

**Figure 5:**
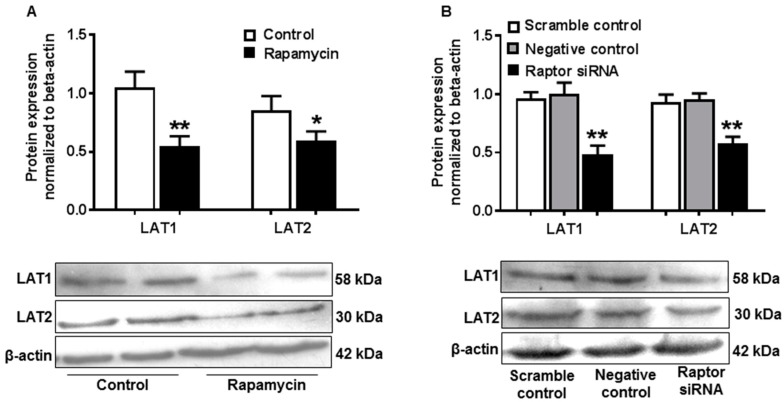
Inhibition of mTORC1 activity resulted in decreased amino acid transporter expression in JEG-3 trophoblast cells. (**A**) Protein expression of LAT1 and LAT2 assayed by Western blot in JEG-3 cells treated with or without rapamycin. The bar graphs show relative expression after being normalized with β-actin expression in each sample. Rapamycin induced a significant decrease in LAT1 and LAT2 expression. Data are from five independent experiments. * *p* < 0.05, **p < 0.01, control versus rapamycin. (**B**) Expression of LAT1 and LAT2 detected by Western blot in JEG-3 cells treated with scrambled or negative or raptor siRNA. The bar graph shows data from five independent experiments. Raptor siRNA caused a significant reduction in LAT1 and LAT2 expression. ** *p* < 0.01, scrambled control or negative control versus raptor siRNA.

To determine the effects of decreased mTORC1 activity on amino acid transporter in placental trophoblast cells, rapamycin, a specific mTORC1 inhibitor, was employed to inhibit mTORC1 activity in JEG-3 cells. Expression of LAT1 and LAT2 was measured by Western blot. As shown in Figure 5A, LAT1 (1.05 ± 0.14 vs. 0.55 ± 0.08, control vs. rapamycin, *p* < 0.01) and LAT2 (0.85 ± 0.12 vs. 0.60 ± 0.08, control vs. rapamycin, *p* < 0.05) expressions were both significantly reduced in rapamycin-treated cells compared with control cells.

mTORC1 inhibition was also conducted by gene silencing targeting raptor, a key component of mTORC1. Consistently, protein expressions of LAT1 and LAT2 were significantly decreased in cells treated with raptor small interfering RNAs (siRNAs) compared to scrambled and negative control cells (Figure 5B).

**Figure 6:**
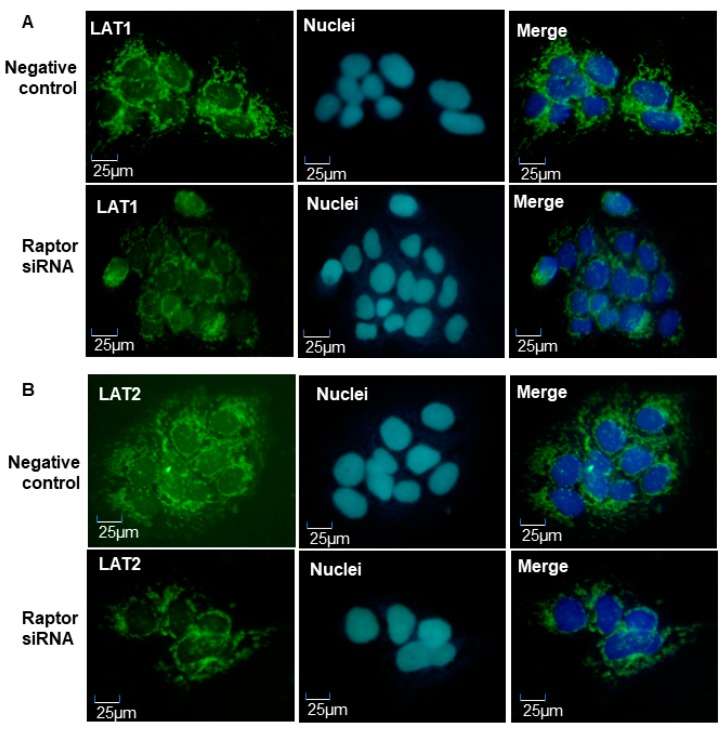
Representative imaging of immunofluorescent staining of LAT1 and LAT2 in JEG-3 trophoblast cells treated with negative or raptor siRNA. (**A**) LAT1 expression; (**B**) LAT2 expression. Consistent with Western blot results, raptor siRNA could reduce LAT1 and LAT2 expression in placental trophoblast.

LAT1 and LAT2 expression was also examined by immunofluorescence staining in JEG-3 cells treated with raptor siRNAs. Consistent with Western blot data, LAT1 (Figure 6A) and LAT2 (Figure 6B) expressions were reduced in cells cultured with raptor siRNAs compared to negative controls. Our results suggested that amino acid transporter expression was regulated by placental mTORC1 signaling pathway, and that inhibition of mTORC1 activity could lead to decreased expression of amino acid transporter in human trophoblast cells.

## 3. Discussion

In the present study, we obtained interesting findings showing that fetal weight and fetal/placental weight ratio was significantly decreased in a rat model of STZ-induced diabetes compared to normal pregnancies, which confirmed that pregestational diabetes could lead to placental dysfunction and fetal intrauterine growth restriction.

The influence of maternal diabetes on fetal and placental growth might be related to the degree of gestational glucose intolerance, which could lead to opposite fetal outcomes. In some studies, an increased birthweight and placental weight was found in pregnancies complicated with mild diabetes [9], but limited fetal intrauterine growth, miscarriage stoics, and malformations were usually reported in pregnancies with severe diabetes [10], which was verified in our study. Weiss’s study found that hyperglycemia in vitro could inhibit the proliferation of first-trimester trophoblast cells [11], which suggested that a reduced growth of the placenta might cause the fetal intrauterine growth delay in diabetes.

Placental efficiency, which is defined as fetal/placental weight ratio, is a useful predictor of those placentas that have adapted their nutrient transfer per gram placenta. A smaller ratio usually indicates that the placenta is less functionally efficient. Several studies have found that fetal/placental weight ratio was reduced in rodent models of IUGR [12,13,14], suggesting an inefficient placenta that failed to adapt its nutrient supply to meet the demands of the growing fetus. Additionally, it has been observed that fetal/placental weight ratios are associated with a variety of human pregnancy syndromes, including preeclampsia, preterm birth, and IUGR [15,16]. Therefore, our findings provide evidence that decreased placental efficiency plays important role in the fetal growth delay in pregestational diabetes mellitus.

Amino acids are crucial for the development of fetal tissue organs, and active transport of amino acid via placental amino acid transporters is the only way for fetuses to obtain amino acids from maternal circulation. Decreased expression or activity of the placental amino acid transporter system has been found in human IUGR or animal models [2,17], but few studies have been performed to investigate the alterations of placental amino acid transporters in pregnancies complicated with diabetes. Our results showed that the expression of placental amino acid transporters was reduced in the rat model of severe diabetes. In addition, using JEG-3 cell line as a human trophoblast cell model, we verified that the hyperglycemia in vitro could down regulate the expression of amino acid transporters in human trophoblast. These findings suggested that restricted fetal growth resulting from maternal diabetes was associated with the decreased placental amino acid transporter expression. Kuruvilla et al. found that the number of system A amino acid transporters on placental microvillus membrane (MVM) was decreased in diabetic pregnancies associated with macrosomia [18]. In contrast, Jansson et al. reported an increased activity of system A amino acid transporters on the placental MVM, regardless of the type of gestational diabetes [19]. The study methods and subjects may have contributed to the difference in the results. Recently, Ericsson et al. found that glucose injection during early gestation in rats had no significant effects on the expression of placental system A amino acid transporter, but that the transport activity of amino acid transporter was significantly decreased [20].

mTORC1 signaling pathway has been considered as a nutrient sensor in the placenta. It has been well studied that placental mTORC1 activity was decreased in IUGR model [6,21], but few studies were taken to investigate the placental mTROC1 changes in pregnancies complicated with diabetes. Our results found that the activity of placental mTORC1 was significantly reduced in a rat model with severe pregestational diabetes. Furthermore, the in vitro study performed in JEG-3 trophoblast cells confirmed that the high glucose could reduce the activity of mTORC1 signaling pathways. These results provided direct evidence revealing the alterations of placental mTORC1 pathway in diabetic pregnancies. Several studies have reported the mTORC1 changes in people with obesity, which is one of the high risks of diabetes. For example, some studies found that mTORC1 activity was promoted in obese people because of high insulin levels or increased inflammatory cytokines [22]. In contrast, Jansson et al. found that obesity could inhibit the activity of placental mTORC1 in a mouse model of obesity [23].

To determine the effects of decreased mTORC1 activity on placental amino acid transporters, we combined rapamycin treatment and gene silencing targeting raptor to inhibit mTORC1 signaling in JEG-3 cells. Our results showed that expression of system L amino acid transporters was reduced in trophoblast, of which mTORC1 signaling pathway was suppressed. It suggested that trophoblast mTORC1 could modulate amino acid transfer across the placenta by regulating the expression of key amino acid transporters. In contrast to Rosario’s report that trophoblast mTORC1 regulation of amino acid transporters occurred mainly by modulation of the translocation of specific transporter isoforms between plasma membrane and cell interior [5], our results showed that mTORC1 could regulate the trophoblast amino acid transporter at the translational level.

In summary, our findings have significant impact on abnormal fetal growth in diabetic pregnancies. In the present study, we found that inefficient placenta was associated with limited fetal growth in a rat model of severe gestational diabetes. Furthermore, we found that placental amino acid transporter expression and mTORC1 activity was reduced in this model. We also demonstrated that decreased mTORC1 activity could lead to decreased amino acid transporter expression in placental trophoblast. These results provide evidence that decreased placental mTORC1 activity could lead to reduced placental amino acid transporter expression and, subsequently, contribute to limited fetal growth in severe diabetic pregnancies.

## 4. Materials and Methods

### 4.1. Animals

Adult female Sprague Dawley rats were given with a single intraperitoneal injection of streptozotocin (STZ) (65 mg/kg) to induce diabetes. At 3 days after STZ injection, plasma glucose concentrations were measured using a glucose analysis kit. The diabetic state was defined as a plasma glucose concentration exceeding 19.5 mmol/L (180 mg/dL). Both normal and diabetic females were mated overnight with normal male rats. Pregnancy was confirmed by the presence of sperm in vaginal smears. The same day that sperm appeared in vaginal smears was designated day 0 of gestation. All animals were housed under controlled temperature (23 °C) and a 12 h light/dark cycle, and they had free access to standard rat chow and tap water. The protocols for all animal experiments were approved by the Institutional Animal Care and Use Committee (IACUC) of Harbin Medical University (approval code: QC2017089; approval date: 1 October 2017).

### 4.2. Collection of Blood and Placenta Samples of Rat

Animals were killed by cervical dislocation at gestation days 21. Both offspring and placentas were carefully dissected out and immediately weighed. Thereafter, the placenta samples were immediately frozen in liquid nitrogen and kept at −80 °C until usage.

### 4.3. Human Trophoblast Cell Culture

Human trophoblast cell line JEG-3 was purchased from the cell bank of the Chinese Academy of Sciences. They were cultured in Dulbecco’s modified Eagle’s medium (Hyclone, China) containing 10% FBS and 1% penicillin/streptomycin, and incubated at standard culture conditions of 5% CO_2_ in air at 37 °C.

### 4.4. mTORC1 Inhibition in Placental Trophoblast

Downregulation of mTORC1 activity was conducted as described in a previous study [4]. Briefly, human JEG-3 cells were cultured with 100 nM rapamycin (Gene Operation, USA), a specific mTORC1 inhibitor, or transfected of small interference RNAs (GenePharma, Shanghai, China) targeting raptor (100 nM; sense: 5′-CGAGAUUGGACGACCAAAUTT-3′). At 48 h in culture, cells were collected for RNA and protein extraction.

### 4.5. RNA Isolation and Quantitative Real-Time PCR

Total RNA was extracted from placenta tissue and JEG-3 cells using the trizol reagent (Invitrogen, United Kingdom). cDNA was synthesized using the PrimeScript 1st Strand cDNA Synthesis Kit (Takara, Japan), following the manufacturer’s instructions. Real-time PCRs were performed in 20 µL mixtures using the SYBR Premix Ex TaqTM II Kit (Takara, Japan). Gene expression was assayed in duplicate and normalized against β-actin. Relative expression values were calculated by the ^ΔΔ^CT method of relative quantification using Applied Biosystems 7500 Real-Time PCR System.

### 4.6. Western Blot Analysis

Protein expression of LAT1, LAT2, p-4EBP1 (Thr-37/46), and p-S6k1 (Thr-389) was examined by Western blotting. An aliquot of 20 µg of total protein was subject to electrophoresis then transferred to nitrocellulose membrane. After blocking, the membranes were probed with anti-human antibody against LAT1, LAT2, p-4EBP1 (Thr-37/46), or p-S6k1 (Thr-389) and followed by corresponding biotinylate conjugated secondary antibodies. Antibodies against LAT1 and LAT2 were obtained from Santa Cruz Biotechnology; p-4EBP1 (Thr-37/46) and p-S6k1 (Thr-389) antibodies were purchased from Cell Signaling Technology (Danvers, MA). The bound antibody was visualized with an enhanced chemiluminescent detection kit (Thermo Scientific). β-actin expression was used as loading control for each sample. The band densities were scanned and analyzed by Alpha Imager software (Alpha Innotech Corporation, CA, USA).

### 4.7. Immunohistochemistry

Expression of LAT1 and LAT2 was also examined by immunohistochemistry (IHC) staining in paraffin-embedded placental tissue sections. A standard IHC staining procedure was performed as previously described [24]. Briefly, a series of deparaffinization was carried out with xylene and ethanol alcohol. Antigen retrieval was performed by boiling tissue slides with 0.01 mol/L citric buffer. Hydrogen peroxide was used to quench the endogenous peroxidase activity. After blocking, the sections were incubated with primary monoclonal antibodies specific against human LAT1 or LAT2 overnight at 4 °C. Corresponding biotinylate conjugated secondary antibodies and ABC staining system were subsequently used, according to the manufacturer′s instructions. Slides stained with the same antibody were all processed at the same time. Stained slides were reviewed under an Olympus microscope, and images were captured by a digital camera with PictureFrame computer software.

### 4.8. Immunofluorescence

LAT1 and LAT2 expression were examined by immunofluorescence in JEG-3 cells transfected with scramble or raptor siRNA. The cells were fixed with methanol at −20 °C for 5 min. After permeablilized by Triton X-100, cells were blocked in goat serum and then incubated with anti-LAT1 or anti-LAT2 antibody overnight at 4 °C. After several washes, cells were incubated with fluorescein isothiocyanate (FITC)-conjugated goat anti-rabbit IgG for 1 h at 37 °C, and then with dye 4,6-diamido-2-phenylindole (DAPI) for 15 min to counterstain nuclei. After three washes in PBS, coverslips were mounted in a drop of 30% glycerol. Images were acquired with a NikonE800 fluorescence microscope.

### 4.9. Data Presentation and Statistics

Data were presented as means ± SD. Statistical analysis was performed with unpaired *t*-test or AVOVA using GraphPad Prism 7 software. A probability level of less than 0.05 was considered statistically significant.

## Figures and Tables

**Table 1:** Data of maternal glucose concentration, fetal and placental weight, and fetal/placental weight ratio in normal and STZ-D rats.

Group	Normal	STZ-D	*n*	*p*-Value
Maternal glucose concentration (mg/dL)	103 ± 29.78	395 ± 118.3	13	<0.001
Fetal weight (g)	4.57 ± 0.95	2.19 ± 1.21	30	<0.001
Placental weight (g)	0.62 ± 0.11	0.65 ± 0.17	30	0.42
Fetal/placental weight ratio	7.60 ± 2.01	5.11 ± 1.59	30	<0.001

The data are shown as mean ± standard deviation (SD).
